# A non-invasive system to monitor in vivo neural graft activity after spinal cord injury

**DOI:** 10.1038/s42003-022-03736-8

**Published:** 2022-08-10

**Authors:** Kentaro Ago, Narihito Nagoshi, Kent Imaizumi, Takahiro Kitagawa, Momotaro Kawai, Keita Kajikawa, Reo Shibata, Yasuhiro Kamata, Kota Kojima, Munehisa Shinozaki, Takahiro Kondo, Satoshi Iwano, Atsushi Miyawaki, Masanari Ohtsuka, Haruhiko Bito, Kenta Kobayashi, Shinsuke Shibata, Tomoko Shindo, Jun Kohyama, Morio Matsumoto, Masaya Nakamura, Hideyuki Okano

**Affiliations:** 1 Department of Orthopaedic Surgery, Keio University School of Medicine, 35 Shinanomachi, Shinjuku-ku, Tokyo, 160-8582 Japan; 2 Department of Physiology, Keio University School of Medicine, 35 Shinanomachi, Shinjuku-ku, Tokyo, 160-8582 Japan; 3 Laboratory for Cell Function and Dynamics, Brain Science Institute, RIKEN, 2-1 Hirosawa, Wako, Saitama, 351-0198 Japan; 4 Laboratory for Molecular Analysis of Higher Brain Function, Brain Science Institute, RIKEN, 2-1 Hirosawa, Wako, Saitama, 351-0198 Japan; 5 Department of Neurochemistry, Graduate School of Medicine, The University of Tokyo, 7-3-1 Hongo, Bunkyo-ku, Tokyo, 113-0033 Japan; 6 Section of Viral Vector Development, National Institute for Physiological Sciences, 38 Nishigonaka Myodaiji, Okazaki, Aichi 444-8585 Japan; 7 Division of Microscopic Anatomy, Graduate School of Medical and Dental Sciences, Niigata University, 1-757 Asahimachi-dori, Chuo-ku, Niigata, Niigata, 951-8510 Japan; 8 Electron Microscope Laboratory, Keio University School of Medicine, 35 Shinanomachi, Shinjuku-ku, Tokyo, 160-8582 Japan

**Keywords:** Neural stem cells, Regeneration

## Abstract

Expectations for neural stem/progenitor cell (NS/PC) transplantation as a treatment for spinal cord injury (SCI) are increasing. However, whether and how grafted cells are incorporated into the host neural circuit and contribute to motor function recovery remain unknown. The aim of this project was to establish a novel non-invasive in vivo imaging system to visualize the activity of neural grafts by which we can simultaneously demonstrate the circuit-level integration between the graft and host and the contribution of graft neuronal activity to host behaviour. We introduced Akaluc, a newly engineered luciferase, under the control of enhanced synaptic activity-responsive element (E-SARE), a potent neuronal activity-dependent synthetic promoter, into NS/PCs and engrafted the cells into SCI model mice. Through the use of this system, we found that the activity of grafted cells was integrated with host behaviour and driven by host neural circuit inputs. This non-invasive system is expected to help elucidate the therapeutic mechanism of cell transplantation treatment for SCI.

## Abstract (web summary)

Visualisation of the activity of neural grafts using engineered luciferase provides insights into the integration between the graft and host.

## Introduction

Spinal cord injury (SCI) results in severe neurological dysfunction, including motor, sensory, and autonomic paralyses. In recent years, many attempts to develop cell transplantation therapies to promote regeneration of the damaged spinal cord have been made. Neural stem/progenitor cells (NS/PCs) are some of the most promising resources for such therapies^1–3^. Several putative underlying mechanisms have been suggested, including cell replacement by grafted NS/PC-derived neurons, astrocytes, and oligodendrocytes; trophic support; and axonal remyelination^4–6^. Furthermore, several studies have proposed that NS/PC grafts can form neuronal relays across sites of spinal transection^7–9^, that is, combine input from the rostral part of the host to the graft and output from the graft to the caudal part; these processes are thought to play a major role in functional recovery. However, a detailed characterization of neuronal relay has not been carried out, and how the graft functionally integrates into the host neural circuit is poorly understood. This is mainly because no current technologies can directly monitor the relationships between graft cell activity and the behaviours and circuit-level activities of the host. To elucidate functional host–graft coordination and to evaluate how the graft influences host neural circuit activity and host behaviour, a novel non-invasive in vivo imaging technique to monitor the activity of graft neurons over time within the living host is needed.

To realize such an in vivo monitoring system, we focused on two novel technologies. The first was the AkaBLI system (a combination of the AkaLuc enzyme and AkaLumine-HCl as a substrate with high permeability)^10,11^. Bioluminescence imaging (BLI) is a non-invasive method for measuring light output from cells expressing the enzyme luciferase after luciferin (substrate) administration in living animals^12^. AkaBLI is a newly developed redshifted BLI system that produces bright emission spectra and enables deep tissue imaging in living animals^10^, which is the most appropriate for widefield non-invasive monitoring of gene expression from graft cells in injured spinal cords. The second was enhanced synaptic activity-responsive element (E-SARE), a potent neuronal activity-dependent synthetic promoter^13^. When a neuron becomes active, it switches on immediate-early genes (IEGs), such as *Fos*, *Arc*, and *Egr1*, even in spinal cord neurons, and the promoters/enhancers of IEGs are used as activity-dependent reporter systems^14,15^. Among these promoters is the synthetic promoter E-SARE, which is based on the SARE enhancer element of the *Arc* promoter and drives neuronal activity-dependent gene expression significantly superior to that of any other existing IEG promoters.

In this study, we combined AkaBLI and E-SARE technology and established a novel non-invasive system to visualize the neuronal activity of the graft in vivo. We succeeded in imaging the active ensemble dynamics of NS/PC-derived cells grafted in injured spinal cords. Using this system, we confirmed that graft activity is linked to host behaviour and that the host circuit regulates graft activity.

## Results

### The ESAL system: neuron activity monitoring by bioluminescence

**Fig. 1:**
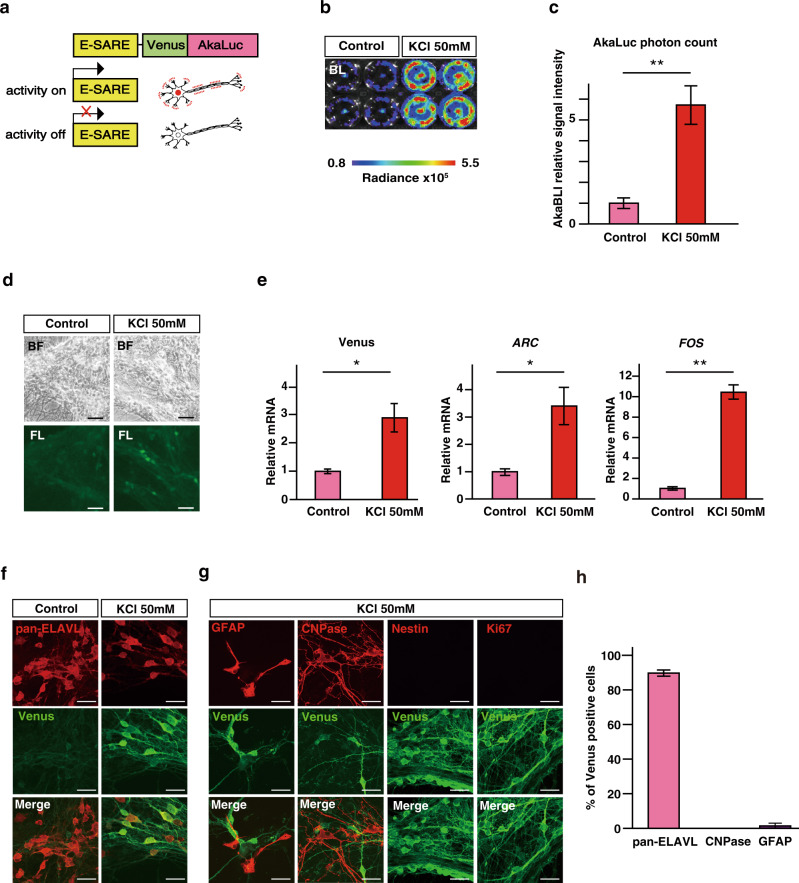
BLI of NS/PC-derived cells stimulated with high potassium in vitro. **a** Schematic illustration of the E-SARE-Venus-AkaLuc (ESAL) construct, which was used to express the Venus-fused AkaLuc luminescent enzyme under the control of the promoter E-SARE. When a neuron was activated, the promoter E-SARE drove high expression of the downstream reporter gene Venus-AkaLuc. **b** Comparative bioluminescence imaging (BLI) of in vitro-cultured cells stimulated with 50 mM KCl for 6 h (on the right, *n* = 4) or without stimulation (on the left, *n* = 4) on the left. We prepared *n* = 8 wells from two independent tertiary neurospheres from the same embryoid body (EB). The colour of the bars indicates the total bioluminescence radiance (photons/sec/cm^2^/str). The steradian (str) is the unit of the solid angle. **c** Quantitative analyses of the relative BLI signal intensity of ESAL-NS/PC-derived cells with or without the addition of 50 mM KCl in vitro (*n* = 4 each). The values are the mean ± standard error of the mean (SEM): **p < 0.01. A two-sided unpaired Student’s *t* test was performed. *T* value and degrees of freedom: t (6) = 11.59, *p* = 2.5 × 10^−5^. **d** Microscopic bright-field image and Venus fluorescence image of ESAL-NS/PC-derived cells with or without the addition of 50 mM KCl in vitro. Scale bar, 50 μm. **e** The results of qPCR analyses of the gene expression of Venus, *ARC*, and *FOS* in cells within the same well as shown above (*n* = 4 each). The values are the mean ± SEM: *p < 0.05, **p < 0.01. A two-sided unpaired Student’s *t* test was performed. Individual *t* values and degrees of freedom: Venus *t*(6) = 3.034, *p* = 0.023. Arc *t*(6) = 2.705, *p* = 0.035. Fos *t*(6) = 7.057, *p* = 4.1 × 10^−4^. **f**, **g** Representative images of Venus-expressing differentiated cells from NS/PCs stained for panembryonic lethal abnormal vision-like (pan-ELAVL) (neurons) with or without the addition of 50 mM KCl (**f**); stained for human glial fibrillary acidic protein (GFAP) (astrocytes), 2ʹ, 3ʹ -cyclic nucleotide 3ʹ-phosphodiesterase (CNPase) (oligodendrocytes), Nestin and Ki-67 (immature cells) with the addition of 50 mM KCl (**g**). Scale bar, 20 μm. **h** Percentage of cells positive for cell type-specific markers among the Venus+ cells after stimulation with 50 mM KCl.

To establish a bioluminescence-based system to visualize neuronal activity, we first constructed a lentiviral vector for the expression of AkaLuc, a luciferase optimized for redshifted bioluminescence, under the control of E-SARE, a potent neuronal activity-dependent promoter generated from *Arc* enhancer elements (Fig. 1a). We termed this system ESAL (E-SARE-AkaLuc). In the ESAL system, we also fused AkaLuc with the Venus protein for simultaneous fluorescent labelling and with the PEST sequence to shorten the half-life of the fusion protein^16^. The Venus protein is an *Aequorea victoria*-derived yellow fluorescent protein (YFP)-containing mutation that causes rapid maturation and increased environmental resistance^17^. We then transfected the ESAL lentiviral vector into human-induced pluripotent stem cell (iPSC)-derived NS/PCs, identified as ESAL-NS/PCs, and induced differentiation of the cells into neurons (Fig. 1b–h). When stimulated by a depolarizing concentration of potassium chloride (50 mM), ESAL-NS/PC-derived neurons showed significant increases in AkaLuc photon counts compared with those of unstimulated Controls (Fig. 1b, c). We also detected increases in Venus fluorescence and IEG expression upon 50 mM KCl stimulation (Fig. 1d, e). Thus, we confirmed that the ESAL system was highly sensitive to depolarizing stimulation in neurons but showed little response in nonneuronal cells (Fig. 1f–h). These data suggest that the ESAL system can be used to successfully monitor the neuronal activity of NS/PC-derived neurons.

To profile the temporal resolution of the ESAL system, we examined the time course of bioluminescence after a brief bout of neuronal stimulation (4-Amynopyridine [4AP] + bicuculline [BIC]) that facilitates action potential firing and potentiates glutamatergic transmission (Supplementary Fig. 1a). Neuronal activity-dependent bioluminescence was detected approximately 4 h after stimulation, reached its peak at 6 h, and returned to the basal level at 24 h (Supplementary Fig. 1b). These results indicate that an increase in bioluminescence in the ESAL system reflects cumulative neuronal activity that persisted during a period ranging from 4 to 10 h prior to measured BLI measurement.

### Monitoring the activity of neuronal grafts in an SCI model by ESAL

**Fig. 2:**
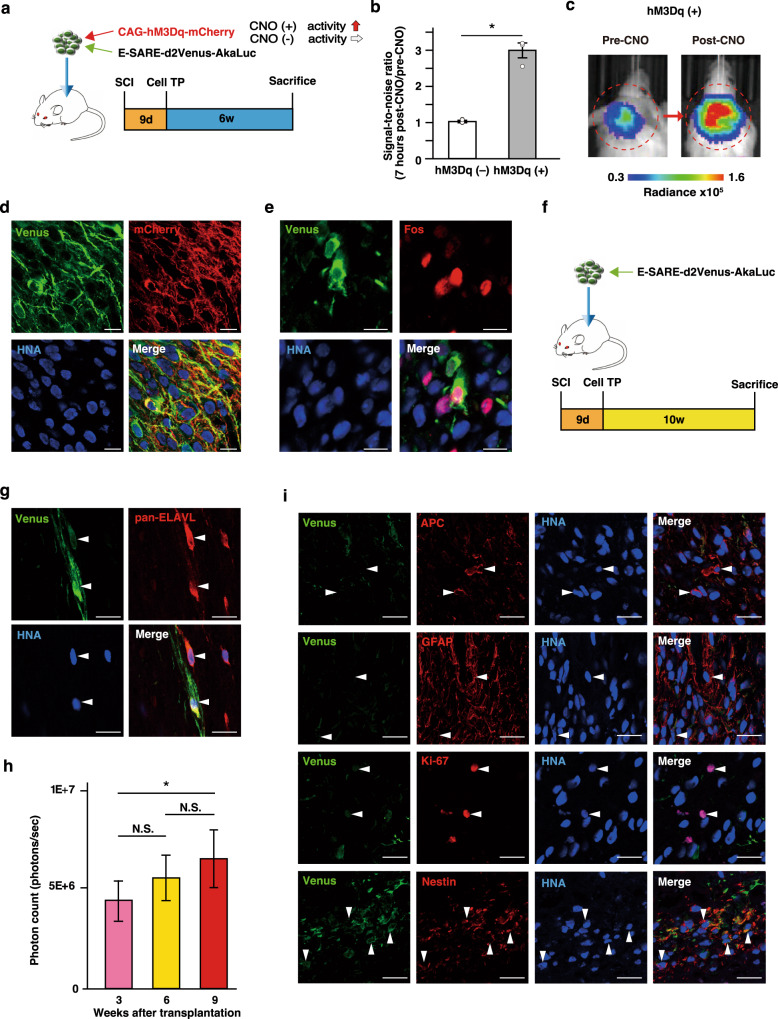
In vivo application of ESAL-expressing NS/PC transplantation after cervical spinal cord injury. **a** Schematic illustration of the positive control mice. Transplantation of NS/PCs double-infected with the two viruses (CAG-hM3Dq-mCherry, which contains an hM3Dq and mCherry fusion protein, and ESAL were transduced into NS/PCs via lentivirus) was performed 9 days after C5 dorsal column transection. Six weeks after transplantation, luminescence measurements were performed before sacrifice. **b** Comparison of the ratios of BLI signal intensity (7 h post-CNO/pre-CNO) between ESAL-expressing NS/PC-transplanted (ESAL-NS/PC-transplanted) mice (hM3Dq [−] TP, *n* = 4) and double-infected NS/PC-transplanted mice (hM3Dq [+] TP, *n* = 3) are shown. The values are the mean ± SEM: *p < 0.05. A two-sided unpaired Student’s *t* test was performed. *T* value and degrees of freedom: *t*(5) = 3.977, *p* = 0.011. **c** Representative IVIS images of a positive control mouse (pre- and post-CNO). The circle shows the region of interest (ROI) in the cervical spine. The colour of the bars indicates the total bioluminescence radiance (photons/sec/cm^2^/str). **d**, **e** Representative images from a positive control mouse 6 weeks after transplantation; labelled with Venus (green), mCherry (red), and HNA (human cells) (blue) (**d**) or labelled with Venus (green), Fos (red), and HNA (blue) (**e**). Scale bars, 20 μm. **f** Schematic illustration of in vivo experiments. Transplantation of ESAL-NS/PCs was performed 9 days after C5 dorsal column transection. Three, 6, and 9 weeks after transplantation, luminescence measurements were performed. All mice were sacrificed 10 weeks after transplantation. **g** Representative images of grafted cells from an ESAL-NS/PC-transplanted mouse labelled with Venus (green), pan-ELAVL (red; arrowheads), and HNA (blue). Scale bars, 20 μm. **h** Time-dependent change in graft luminescence intensity of ESAL-NS/PC-transplanted mice at 3, 6, and 9 weeks after transplantation (*n* = 12 mice). The values are the mean ± SEM: *p < 0.05. N.S.: not significant. A repeated-measures ANOVA was performed. Individual *p* values: weeks 3 and 6; 0.1735, weeks 6 and 9; 0.2007, weeks 3 and 9; 0.0035. **i** Representative images of grafted cells from ESAL-NS/PC-transplanted mouse tissues labelled with Venus (green), APC (oligodendrocytes), GFAP (astrocytes), Ki-67/Nestin (red; arrowheads), and HNA (blue). Scale bars, 20 μm.

Next, NOD/ShiJic-scidJcl (NOD-SCID) mice were subjected to spinal level C5 dorsal column transection and to transplantation 9 days after injury. We considered the host environment to be most suitable for transplantation at this time because acute inflammation after SCI has subsided, while glial scar formation is not yet completed^18–21^. ESAL-NS/PCs were transplanted into the injury sites (Fig. 2a, f). We found that grip strength normalized by body weight and the IBB score had improved significantly more in the TP group than in the PBS group (Supplementary Fig. 2a, b). However, no significant changes related to recovery were found in the error rate for the horizontal ladder test (Supplementary Fig. 2c).

To artificially manipulate the neuronal activity in the grafted cells, we introduced hM3Dq, a stimulatory chemogenetic receptor and designer receptor exclusively activated by designer drugs (DREADDs), which enabled the graft to be activated upon administration of its ligand^22,23^ into ESAL-NS/PCs (Fig. 2a). When the grafts were activated by administration of the hM3Dq ligand clozapine N-oxide (CNO), bioluminescence was significantly upregulated (Fig. 2b, c). Consistently, in immunohistochemical analyses, Venus protein expression was detected exclusively in hM3Dq-expressing cells, while mCherry+ graft cells mostly expressed Venus protein upon CNO activation (Fig. 2d). To confirm that this occurred because the lentiviral transfection efficacy was very high, we calculated the transfection efficiency of ESAL and DREADD into NS/PCs via a lentiviral vector in vivo. First, the transfection efficiency of hM3Dq under a ubiquitous promoter, that is, the population of mCherry+ cells out of human neutrophil antigen (HNA)+ cells, was 90.0 ± 1.1% (Fig. 2d, Supplementary Fig. 2d) (from *n* = 3 animals). Second, the expression efficiency of ESAL was approximated according to the population of Venus+ mCherry+ double-positive cells out of mCherry+ cells under ubiquitous CNO activation. The Venus+mCherry+/mCherry+ percentage was determined to be 83.9 ± 2.5% (Fig. 2d, Supplementary Fig. 2d) (from *n* = 3 animals). Overall, the transfection efficiencies were considered to be high enough to interpret the results of bioluminescence. Consistent with this, Venus+ cells were often immunopositive for Fos, a marker and an IEG (68.9 ± 3.0%) (Fig. 2e), even though the Fos protein is immediately upregulated by neuronal activity and is a short-lived transcription factor^24,25^. These data suggested that the ESAL system successfully labelled active ensembles in neurons grafted into the SCI model mice.

The percentage of human ELAVL (Hu)-positive neuronal cells was very high among the Venus− positive cells (76.2 ± 8.6%) (Supplementary Fig. 2e). Even without artificial activation by hM3Dq, we found Venus expression in a portion of neuronal grafts (Fig. 2f, g). This finding suggests that the ESAL system may report neurons showing spontaneous activity in the graft. This is in line with the time course of ESAL bioluminescence elevation measured chronologically after ESAL-NS/PC transplantation, suggesting that a progression of neuronal differentiation and maturation of NS/PCs precedes a significant increase in bioluminescence detection (Fig. 2h). Consistent with this idea, we confirmed that few glial cells exhibited Venus expression (GFAP+/Venus+, 4.0 ± 1.5%; APC+/Venus+, 3.3 ± 2.5%) (Fig. 2i).

### Graft neurons integrate into the host nervous system

**Fig. 3:**
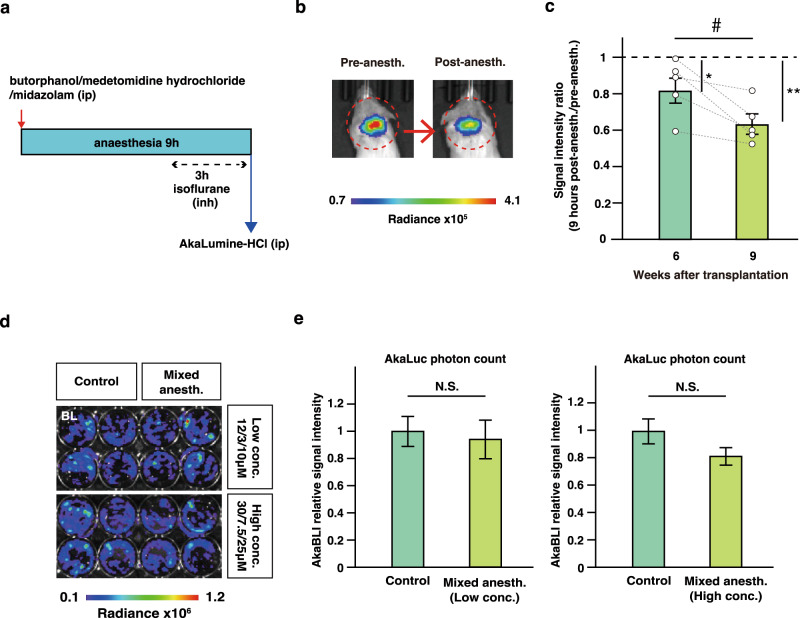
Continuous anaesthetization of ESAL-expressing NS/PC-transplanted mice. **a** Schematic illustration of long-term continuous anaesthesia. Anaesthesia was achieved with a mixture of three types of anaesthetic agents (butorphanol, medetomidine hydrochloride, and midazolam) followed by inhalation anaesthesia for up to 9 h. **b** Representative IVIS images of an ESAL-NS/PC-transplanted mouse before and after long-term continuous anaesthesia 10 weeks after transplantation. The circle shows the region of interest (ROI) in the cervical spine. The colour of the bars indicates the total bioluminescence radiance (photons/sec/cm^2^/sr). **c** The ratio of BLI signal intensity (pre- and 9 h post-continuous anaesthesia) at 6 and 9 weeks after transplantation (*n* = 5 mice). The values are the mean ± SEM: *p, ^#^*p* < 0.05. Two-sided paired Student’s *t* tests were performed. Individual *t* values and degrees of freedom: week 6 and 9; *t*(8) = 2.754, *p* = 0.025, week 6; t(8) = 2.502, *p* = 0.037, week 9; *t*(8) = 4.456, *p* = 2.1 × 10^−3^. **d** Comparative BLI of in vitro-cultured cells with (*n* = 4) or without (*n* = 4) the addition of the mixed anaesthetic agents at two different concentrations, 12/3/10 μM (“Low concentration”) and 30/7.5/25 μM (“High concentration”), for 6 h (*n* = 4, 4 each). We prepared *n* = 8 wells from two independent tertiary neurospheres from the same EB. The colour of the bars indicates the total bioluminescence radiance (photons/sec/cm^2^/str). **e** Quantitative analyses of the relative BLI signal intensity of ESAL-NS/PC-derived cells with or without the addition of the mixed anaesthetic agents in vitro (*n* = 4, 4 each). The values are the mean ± SEM: N.S.: not significant. Two-sided unpaired Student’s *t* tests were performed. Individual *t* values and degrees of freedom: left: *t*(6) = 0.342, *p* = 0.744, right: *t*(6) = 1.821, *p* = 0.118.

Previous reports have suggested that after injury to the host neuronal tract, the grafted neurons integrate and become part of the circuitry within the tract^9,26^. Using the ESAL system, we examined the effect of host activity on neuronal graft activity at the individual and circuit levels. First, we monitored ESAL bioluminescence over the day and found that ESAL bioluminescence was highest around noon and lowest at night (Supplementary Fig. 3). Given that ESAL bioluminescence reflects cumulative neuronal activity approximately 6 h before observation (Supplementary Fig. 1 a, b), this result indicates that graft neuronal activity has diurnal variations consistent with the peak and trough activity of the host animals during the night and day periods, respectively. To further determine how much host activity influences graft activity at the individual level, we next utilized long-term anaesthesia (with a combination of midazolam, medetomidine hydrochloride, and butorphanol) to mimic sleep (Fig. 3a). The respiration rate of the mice suggested that the anaesthesia was active for at least 6 h following administration. To increase the anaesthetic time to 9 h, we additionally administered isoflurane for the remaining 3 h. The ESAL bioluminescence decreased to nearly half of the initial level after long-term anaesthesia (Fig. 3b, c). This decrease was significant at both 6 and 9 weeks post-transplantation (Fig. 3c). Additionally, we sought to confirm that the mixture of three types of anaesthetic agents could not directly alter the activity of graft neurons using in vitro-cultured neurons. Indeed, BLI signal intensity was not notably reduced by the anaesthetic agents (Fig. 3d, e). Taken together, these data imply that graft neuronal activity is associated with the daily activities of hosts on an individual level.

### Host neural circuit inputs regulate graft activity

**Fig. 4:**
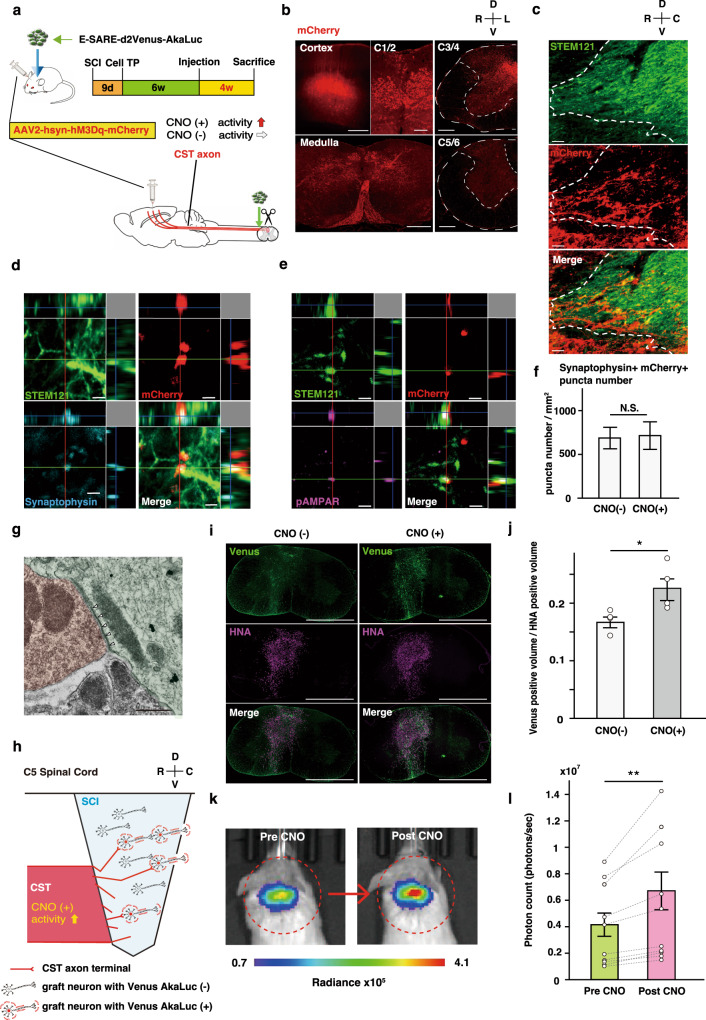
CST stimulation via clozapine N-oxide (CNO) administration through the DREADD system in ESAL-NS/PC-transplanted mice. **a** Schematic illustration representing the time schedule of the in vivo experiments. Six weeks after transplantation of ESAL-NS/PCs, mice were subjected to AAV injection into the motor cortex. Ten weeks after transplantation, luminescence measurements were performed before sacrifice. **b** Confirmation of mCherry-labelled cell bodies in the cerebral cortex (scale bars, 250 μm) and mCherry-labelled axons in the medulla (scale bars, 500 μm) and cervical spinal cord at the 1/2 level (scale bars, 100 μm). mCherry+ CST axon ends were observed in the grey matter rostral to the C5 lesional area (scale bars, 250 μm), and few CST axons were observed caudal to the lesion (scale bars, 250 μm). R: right, L: left, D: dorsal, and V: ventral side. **c** Representative sagittal images 400 μm from the midsagittal plane around the injured spinal cord. Transplanted cells were stained with STEM121 (a human-specific cytoplasmic marker), and the CST was labelled with mCherry. Dashed line, rostral boundary of the graft, R: rostral, C: caudal, D: dorsal, and V: ventral side. Scale bars, 5 μm. **d** High-magnification view of synapse formation between CST axons and grafted neurons. The presynaptic marker synaptophysin merged with mCherry and was adjacent to a STEM121+ cell. Scale bars, 2 μm. **e** High-magnification view of synapse formation between CST axons and grafted neurons. The postsynaptic marker pAMPAR merged with STEM121 and was adjacent to an mCherry+ cell. Scale bars, 2 μm. **f** Bar graph showing the quantification of the synaptophysin+ synapses merged with mCherry integrated into the STEM121+ area in each transplanted group (CST activation group, 20 images/*n* = 4; CST nonactivation group, 20 images/*n* = 4). The values are the mean ± SEM: *p < 0.05. A two-sided unpaired Student’s *t* test was performed. *T* value and degrees of freedom: *t*(38) = 0.128, *p* = 0.90. **g** Double immunoelectron microscopic image of the synaptic connection between an mCherry+ CST neuron with 3,3’-diaminobenzidine tetrahydrochloride (DAB) staining and a Venus+ graft neuron with Immunogold staining. Anti-mCherry labelling was localized at the membrane of the CST neuron, and Venus proteins were detectable as black dots, mainly in the cytoplasmic membrane. Arrowheads, postsynaptic density. Scale bar, 500 nm. **h** Schematic illustration demonstrating that the host CST inputs to neural stem cell grafts in sites of C5 spinal cord injury. When CST neurons are activated via hM3Dq, graft neurons interconnected with regenerated CST axons are capable of obtaining the promoter E-SARE, driving high expression of the downstream reporter gene Venus-AkaLuc. R: rostral, C: caudal, D: dorsal, and V: ventral side. **i** Representative axial cervical spinal cord images of Venus and HNA staining in tissue from mice with or without CST activation. The sections shown were derived in the same order from along the rostro-caudal axis. Scale bars, 1000 μm. Venus is a cytoplasmic protein, while HNA is an anti-nuclear antigen. **j** Comparison of the calculated Venus+ volume/HNA+ volume between transplanted groups (CST activation group, *n* = 4; CST nonactivation group, *n* = 4). An anti-GFP antibody was used to label the Venus protein. The values are the mean ± SEM: *p < 0.05. A two-sided unpaired Student’s *t* test was performed. *T* value and degrees of freedom: *t*(6) = 2.573, *p* = 0.042. **k** Representative IVIS images of an ESAL-NS/PC-transplanted mouse before and after CST activation (the same individual shown in Fig. 4i [right]). The circle shows the region of interest (ROI) in the cervical spine. The colour of the bars indicates the total bioluminescence radiance (photons/sec/cm^2^/str). **l** Comparison of BLI signal intensity for ESAL-NS/PC-transplanted mice with or without CST activation for each mouse 10 weeks after transplantation (*n* = 10 mice). The values are the mean ± SEM: **p < 0.01. The two-sided paired Student’s *t* test was performed. *T* value and degrees of freedom: *t*(18) = 2.963, *p* = 8.3 × 10^−3^.

We next investigated whether and to what extent host circuit-level activity directly regulates graft neuronal activity. We focused on the corticospinal tract (CST), one of the main descending circuits that plays pivotal roles in sensorimotor control, and artificially manipulated CST activity by injection of the motor cortex with an adeno-associated virus (AAV) encoding hM3Dq-mCherry under the control of the human synapsin I promoter (Fig. 4a). Three to four weeks after AAV injection^27^, we confirmed that hM3Dq-mCherry permitted efficient anterograde labelling to the C*5* lesion site through the CST (Fig. 4b). This selective labelling of the CST projections to the lesion site suggested that CST fibres innervated the graft (Fig. 4c, Supplementary Fig. 5). Indeed, we found that host-to-graft synapses had formed (Fig. 4d–g, Supplementary Fig. 6a–c), indicative of CST-driven control of the graft. To test this directly, the CST was activated by administration of the hM3Dq ligand CNO, and we detected an activity-induced increase in the expression of Venus fused with AkaLuc in graft cells by immunohistochemical analyses (Fig. 4h–j). Furthermore, the increase in graft activity induced by artificial CST stimulation upon CNO treatment was also confirmed in vivo by BLI measurements through an increase in ESAL photon counts of the graft (Fig. 4h, k, l). These results suggest that host CST inputs innervate and regulate graft activity.

How long after transplantation do CST inputs increase to innervate grafted neurons? To answer this question, the bioluminescence of grafts innervated by CST neurons was measured longitudinally. Interestingly, the signal-to-noise ratio (post-/pre-CST activation) increased significantly from weeks 3 to 9 and from weeks 6 to 9, suggesting that host CST inputs innervate and regulate graft activity, especially after week 6 (Supplementary Fig. 4a).

How is a time-course of ESAL activity performed post-CNO administration? An initial effect of systemic CNO administration on neuronal activity starts after 5–10 min and reaches its peak at 45–50 min after CNO administration^28–30^. Considering that the time lag revealed by this in vitro study was 6 h from the driving of the ESARE promoter to the peak of reporter protein expression of ESAL, all the measurements above were performed 7 h after CNO administration (Supplementary Fig. 4b). For validation, we performed ESAL photon count measurements at 4, 7, and 10 h after CNO administration, tracking the same individual animal on different days. In line with the in vitro study results, the ratio of the average photon count per initial state was the highest at 7 h among the three time points (4, 7, and 10 h), although the difference between the photon count ratios at 4 h and 7 h per initial state was not significant (Supplementary Fig. 4c).

## Discussion

Here, we developed a novel bioimaging system, ESAL, by combining a sensitive and accurate redshifted bioluminescence, AkaBLI, and the neuronal activity-dependent promoter E-SARE. This ESAL system efficiently labels active neurons in neural grafts in the injured spinal cord. By using this non-invasive ESAL imaging system, we have demonstrated the direct association between graft activity and host circuit-/behaviour-level activity.

The results of this study demonstrate that the ESAL system is a non-invasive method for in vivo imaging of graft activity in the injured spinal cord. The ESAL system can image spontaneous neuronal activity and reveal the interaction between the graft and host neural circuits. Although some groups have reported host–graft connectivity in SCI models using electrophysiological^31^ or calcium imaging techniques^9^, their experiments were not performed in the living SCI animals. In contrast, the ESAL system can be used in an entirely non-invasive manner, and we showed in vivo that host activity at the individual level, such as sleep and long-term anaesthesia, directly influences graft activity.

Neuronal relay is a core mechanism through which NS/PC transplantation can be used to treat SCI^9,31,32^. Relay circuits can be established between host descending axons and newly differentiated neurons from transplanted NS/PCs. However, the lack of non-invasive in vivo measurement methods has precluded elucidation of how neural grafts functionally integrate into host circuits and how graft activity contributes to behavioural recovery. Our ESAL system can monitor graft activity in the context of various host behaviours, leading to elucidation of the mechanism of neuronal relay formation and its contribution to functional recovery.

The bioluminescence intensity of NS/PC-derived cells increased continuously until 9 weeks after transplantation, which suggested that spontaneous graft activity increased concomitantly. This is in keeping with a previous study from our group suggesting that more than 6 weeks is required for neuronal maturation in grafts^33^. Several reports using human iPSC-derived brain organoids have also indicated that synapse formation and spontaneous activity begin at 4–6 weeks^34^. Based on others’ findings and our own, the ESAL system results could be reflective of neuronal maturation and synapse formation in vivo.

The debate remains as to which subtypes of NS/PCs with different regional identities are the most appropriate for cell therapy^31,35,36^. The ESAL system will enable determination of the quality and suitability of various NS/PC subtypes integrated into the host. How grafts contribute to further tissue regeneration and motor function restoration has remained elusive. Previous reports have shown that pretreatment with a γ-secretase inhibitor (GSI) promotes the maturation of NS/PC-derived neurons by inhibiting Notch signalling;^21^ furthermore, Nogo receptor antagonists facilitate raphespinal tract regeneration^37^, and some synapse organizers have been suggested to accelerate synapse formation^38^. This system will also be valuable in evaluating the effects of these molecular components on NS/PC grafts and the host circuitry around the injured spinal cord.

Thus, this ESAL system has the potential to reveal the synaptic input contribution from each descending pathway to graft neurons in motor function recovery. The functions of the CST include control of afferent inputs, spinal reflexes and motor neuron activity^39^. Furthermore, the CST is generally recognized as the principal motor pathway for voluntary movements in humans^40^. In rodents, the CST commands only gripping ability involving digital flexors, which largely depends on the dorsal and dorsolateral CST, as well as horizontal ladder or skilled reaching tasks^41,42^. According to previous studies, the rubrospinal or reticulospinal tract may be more important than the CST for motor function in rodents^43,44^. It would be interesting to verify the transplantation site by using this system to enhance the effect of cell therapy.

In conclusion, this study introduces a new in vivo system to monitor grafted cell-derived neurons and provides important information on the importance of the link between graft activity and the host neuronal circuit and behaviour. We demonstrated in vivo that host-to-graft synaptic connectivity was functionally established after NS/PC transplantation for SCI. One way to improve cell therapy would be to further enhance this connectivity. Promoting neuronal maturation or synapse formation might render cell therapy with this system more effective in the future.

### Limitations of study

Both activation of the CST/graft and the recording of graft activity were dependent on the viral expression of hM3Dq and ESAL. Variances in transfection/expression efficiencies could affect analyses of integration of the graft cells within the host CST. Low transfection efficiencies could also make it difficult to interpret results regarding ESAL activity. Although the CST is the main component contributing to motor control, we recognize that a one-time CST stimulation is not sufficient in improving the behaviour of mice given that neuronal plasticity is not supposed to be altered. Instead, a consecutive hM3Dq stimulation of the CST may achieve simultaneous amelioration of circuit/behavioural activities enhancing synaptic activity^45,46^. Furthermore, inactivating CST function by CST-DREADDs would better help provide evidence of the host-to-graft connectivity^47^.

It is important to note that this system does not provide real-time assessment of ongoing neuronal activity but rather needs increased activity over multiple hours to show an effect, reflecting cumulative neuronal activity. One solution to improve temporal resolution is to utilize an indicator of real-time neuronal activity, such as the intracellular calcium concentration. Indeed, a calcium-dependent luciferase system, Orange CaMBI, has been reported^48^. However, this system is based on NanoLuc-furimazine and might not be appropriate for neural grafts due to its low substrate permeability through the blood–brain barrier^49,50^. Another possible solution is the use of another activity-dependent promoter, such as the *Fos* promoter^51^, which is a commonly used activity-dependent promoter that is regulated at a very low level because of the autorepression of *Fos* transcription by the Fos protein^52,53^. Thus, to amplify *Fos* promoter-dependent expression, a tet-inducible system is usually added due to transcriptional activation by tetracycline^10^. However, this *Fos-*tet double-reporter system requires substantial time for gene expression. In contrast, the ESAL system drives reporter expression at a high enough level to be used alone for graft monitoring, achieving gene expression over a relatively short period.

Although we have mainly focused on the host-to-graft connection between the host descending neurons and the graft, further studies are needed to assess the graft-to-host interaction between the graft and the host spinal neurons, such as motor neurons. The ESAL system is available for not only grafts but also host neurons through the use of AAVs, because ESAL can be packaged into a single AAV due to its small size. For example, it would be feasible to detect the activity of spinal motor neurons by transfecting an AAV retrograde viral vector into the neuromuscular junction^54^. Future studies will extend our understanding of the mutual interaction between host and graft.

## Materials and methods

### Lentiviral vector construction

To construct a lentiviral vector for ESAL, the E-SARE promoter (as described in Ref. 10, and available upon request from H. Bito at the Department of Neurochemistry, University of Tokyo Graduate School of Medicine) and Venus-AkaLuc-PEST cDNA (obtained from a plasmid that we received with an MTA from the RIKEN BRC [bioresource centre]) were cloned into the lentiviral vector CSII. To construct a lentiviral vector for ubiquitous DREADD activation, hM3Dq-mCherry cDNA was polymerase chain reaction (PCR)-amplified from pAAV-hSyn-hM3D(Gq)-mCherry (Addgene plasmid #50474) and transferred to the lentiviral vector CSIV with the CAG promoter, which is a hybrid construct consisting of the cytomegalovirus (CMV) enhancer fused with the chicken beta-actin promoter^55^.

### Lentiviral vector preparation

Recombinant lentiviral vectors were produced by transient transfection of three plasmids into HEK 293T cells: pCAG-HIVgp, pCMV-VSV-G-RSV-Rev, and the lentiviral vector CSII-E-SARE-Venus-AkaLuc-PEST or CSIV-CAG-hM3Dq-mCherry^56–58^.

### NS/PC culture and lentiviral transduction

The Centre for iPS Cell Research and Application (CiRA) provided us with human induced pluripotent cells (iPSCs) generated and maintained under good manufacturing practice (GMP)-conditions. NS/PCs were generated from the human iPSC line 414C2^59^ by previously described methods^21,33,60^. Briefly, 414C2 human iPSCs were cultured for 12 days in adhesion culture with mouse embryonic fibroblasts (MEFs). Then, embryoid bodies (EBs) were generated from iPSCs grown in suspension for 30 days. The EBs were then dissociated into single cells using TrypLE Select (Thermo Fisher Scientific, MA, USA) and differentiated in suspension at a density of 1.0 × 10^5^ cells/ml in KBM neural stem cell medium (Kohjin Bio, Saitama, Japan) supplemented with B-27 (Thermo Fisher Scientific), 20 ng/ml FGF-2 (PeproTech, NJ, USA), and 10 ng/ml human leukaemia inhibitory factor (hLIF; Merck KGaA, Hesse, Germany) for 12 days. FGF-2 was added every 3 days, and all cell culture media were changed every 6 days. These primary neurospheres were passaged every 10–14 days by dissociating them in the same manner as described above. After the first passage, lentiviral infection was performed in culture for 3 days, and tertiary neurospheres were used for the following experiments. Considering the calculated transfection efficiency, we adjusted the MOI (multiplicity of infection) values for NS/PCs to 6.7 (ESAL) and 2.7 (hM3Dq) based on the lentiviral titre by quantitative PCR (qPCR) and the total cell number per flask, which was consistent throughout the experiments. Titre determination was performed by qPCR-based lentiviral titre assay according to the manufacturer’s instructions (Takara Bio, Shiga, Japan).

### In vitro neuronal differentiation

Mouse astrocyte-feeder cells, which had been extracted from the E17 mouse cerebral cortex, were plated on 24-well chamber glass slides coated with poly-D-lysine (Sigma–Aldrich). The feeder cells were cultured at 37 °C in 5% CO_2_ and 95% air for 3–7 days (5 × 10^4^ cells/well). Quaternary neurospheres dissociated using TrypLE Select were plated onto chamber glass slides precoated with the astrocyte-feeder layer at a density of 1×10^5^ cells/well. The cells were cultured for 50 days in neuronal maturation medium consisting of Neurobasal Plus Medium (Thermo Fisher Scientific) supplemented with B-27 Plus Supplement (Thermo Fisher Scientific), GlutaMAX (Thermo Fisher Scientific), Culture One Supplement (Thermo Fisher Scientific), and L-ascorbic acid (200 μM) (Sigma–Aldrich). For the neuronal stimulation assay, potassium chloride was added at a concentration of 50 mM and remained in the culture medium for 6 h. For the neuronal silencing assay, a three-drug mixture (butorphanol [Vetorphale], Meiji Seika Pharma Co., Ltd., Tokyo, Japan; medetomidine hydrochloride [Domitor], Nippon Zenyaku Kogyo Co., Ltd., Fukushima, Japan; midazolam, Sandoz K.K., Tokyo, Japan) was added at two concentrations: 12/3/10 μM (low concentration) and 30/7.5/25 μM (high concentration)^61,62^.

### Animals

Eight-week-old female NOD-SCID mice (Oriental Yeast Co., Ltd., Tokyo, Japan) underwent SCI or sham surgery (the weight of the SCI mice before SCI ranged 15.92–20.55 g). To determine the therapeutic effect of transplantation on motor function, 30 animals were assigned to the ESAL-NS/PC transplantation (TP) group, PBS injection (PBS) group, or sham surgery (Sham) group using the random lottery method (i.e., individual pieces of papers with the numbers 1–30 were placed in a box, and pieces of paper were drawn at random for each group: TP group, n = 14; PBS group, n = 12; sham group, n = 4). The TP group was injected with an AAV at week 1 and underwent longitudinal luminescent measurements (the weight of the TP and PBS mice ranged 15.07–19.69 g at week 0, 15.00–20.75 g at week 3, 16.26–22.86 g at week 6, and 17.14–22.43 g at week 9). Additionally, some TP mice (*n* = 12) preliminarily underwent SCI, TP, and AAV injections at 6 weeks after TP. We performed luminescent measurements only in week 10. Both the ESAL and hM3Dq-transduced NS/PC-transplanted mice (hM3Dq [+] TP, *n* = 5) underwent positive control measurements in week 6 (data were available for *n* = 3). In contrast, other TP mice (hM3Dq [−] TP, *n* = 5) underwent negative control measurements in week 6 (data were available for *n* = 4) and an assessment of the influence of long anaesthesia on grafts in weeks 6 and 9. All animal experiments were approved by the Ethics Committee of Keio University and performed in accordance with the Guide for the Care and Use of Laboratory Animals (National Institutes of Health, MD, USA).

### SCI modelling and transplantation

Eight-week-old female NOD-SCID mice were anaesthetized by intraperitoneal injections of ketamine (60 mg/kg) and xylazine (10 mg/kg). No additional analgesia beyond ketamine was administered. Under inhalational anaesthesia using isoflurane (1–2%) and O_2_, the laminal arch of the vertebrae at the C4 level was removed, and the dorsal surface of the dura mater was exposed. A tungsten wire knife (McHugh Milieux, David Kopf Instruments, CA, USA) was inserted 0.6 mm from the dorsal surface and raised 0.5 mm to transect the dorsal column^31^. Nine days after the injury was made, 5 × 10^5^ NS/PCs per 2 μl were transplanted into the lesion area at a rate of 1 μl/min using a metal needle with a 10-μl Hamilton syringe and a stereotaxic microinjector (KDS 310; Muromachi Kikai, Tokyo, Japan) (ESAL-NS/PC-transplanted mice, total *n* = 31; ESAL and hM3Dq-transduced NS/PC-transplanted mice, *n* = 5). The syringe was left at the injection site for 2 min after the injection before being removed. An equal volume of PBS was injected instead into control mice (PBS group mice, *n* = 12). The sham mice underwent laminectomy of the C4 cervical spine (sham group mice, *n* = 4). Body temperature was maintained at 37 °C by placing the mice on a digital heating plate. We intramuscularly administered 12.5 mg/kg ampicillin to animals on the operation day and the day after SCI. We did not perform any intensive care after SCI because the animals were able to access food and water on their own despite the negative influence on forelimb movement. The animals were kept during a 10-week follow-up period and then sacrificed; the period was 6 weeks for ESAL and hM3Dq-transduced NS/PC-transplanted mice (hM3Dq [+] TP; *n* = 3; two animals died before 6 weeks).

For the transplantation experiments, gamma secretase inhibitor (GSI) treatment was applied on the day before transplantation as described previously^21^. Briefly, NS/PCs were cultured with a small-molecule GSI, *N*-[*N*-(3,5-difluorophenacetyl)-L-alanyl]-S-phenylglycine t-butyl ester (DAPT; Sigma–Aldrich, MO, USA), dissolved in DMSO at a final concentration of 10 μM, for one day before transplantation into the injury site.

### Behavioural analyses

The analyses below were performed in weeks 3, 6, and 9. The experimenter was blinded to the treatment groups at all times during the behavioural experiments.

#### Grip strength test

The grip strength test is an accepted way to assess forelimb function. The recovery of motor function following cell transplantation or PBS injection was assessed based on the ability of the animal to exert a pulling force^63–65^ (TP group; *n* = 13, PBS injection group; *n* = 10 at the final time point in week 9). The trial consisted of five separate pulls. The highest and lowest forces were excluded, and the remaining three forces were averaged^66^. The strength measures were also divided by body weight^67,68^. The grip strength test was performed using a digital force gauge (Shimpo, Kyoto, Japan) and wire mesh attachment device (Muromachi Kikai).

#### Irvine, Beatties and Bresnahan (IBB) score

To assess forelimb impairment, particularly CST function, the mouse IBB score was used following the methods of the Irvine, Beatties and Bresnahan food manipulation task with slight modification^69–71^. Briefly, the mice were acclimated to the environment every day for 10 days after SCI. Donut-shaped and honey-flavoured cereal (Honey Nut Cheerios, MN, USA) was given, and the forelimb movements were recorded with a camera (GoPro, CA, USA) at 120 f/s within the home cage (TP group, *n* = 13; PBS injection group, *n* = 10; sham group, *n* = 4 at the final time point in week 9).

#### Horizontal ladder rung walking task

The horizontal ladder (Conduct Science, MA, USA) walking task measured paw placement on irregularly spaced rungs^72,73^. To assess skilled walking, the errors in each crossing were counted. Before SCI, we trained mice using a regular pattern of rungs 20 min a day for five days to habituate them. A camera (GoPro) was positioned at a slight ventral angle to record all four limbs at 60 f/s. The pattern of the irregularly spaced rungs was altered every 3 weeks to prevent animals from learning the pattern and compensating for impairments through learning. The start was defined as the time when the mouse placed all four limbs on the rungs, and the end was defined as the time when the mouse reached the last rung of the ladder. The first step after interruption was not scored.

### Anterograde labelling and activation of the CST

To rigorously investigate whether CST activation altered the activity of neural grafts in week 10, AAV2-hsyn-hM3Dq-mCherry (Addgene #50474-AAV2; 7.38 × 10^12^ vg/ml) was injected into the bilateral sensorimotor cortex at four sites (500 nL/point; coordinates = 1 mm rostral and 1.4 mm lateral to the bregma, 1 mm posterior and 1 mm lateral to the bregma; depth = 0.7 mm) at a rate of 100 nL/minute through a pulled glass micropipette (calibrated micropipette, 1–5 μL; Funakoshi, Tokyo, Japan) at week 6 (preliminary ESAL-NS/PC-transplanted mice [*n* = 12]). To investigate the association between upper limb movement and the alteration of graft activity after CST activation, AAV2-hsyn-hM3Dq-mCherry was injected into the bilateral sensorimotor cortex at two sites (500 nL/point; coordinates = 1 mm rostral and 1.4 mm lateral to the bregma; depth = 0.7 mm) at week 1 (ESAL-NS/PC-transplanted mice for longitudinal luminescent measurements [*n* = 14]). CNO (Enzo Life Sciences, NY, USA) was intraperitoneally administered at a concentration of 5 mg/kg. To manipulate the CST, a total of 26 ESAL-NS/PC transplanted mice underwent anterograde labelling of CST axons with hM3Dq. To be included in analyses for integration between the host CST and graft, the CST axons had to be successfully transected and labelled according to immunohistochemistry, and the graft had to be clearly detected on the cervical spine by BLI photon counting (at weeks 3, 6, and 9). Out of the preliminary 12 animals, one animal was excluded because of death before final measurement, and one animal was excluded because of poor CST labelling (*n* = 10 available). Of the longitudinal 14 animals, two animals were excluded because of death, and two animals were excluded because of poor CST labelling (*n* = 10 available). CST transection was considered successful for all other mice according to the immunohistochemical findings^74^.

### Luminescence measurement

Bioluminescence images were acquired using the IVIS Spectrum system (Perkin Elmer, MA, USA). For in vitro-cultured neurons, bioluminescence was measured immediately after treatment with 300 µM AkaLumine-HCl (FUJIFILM Wako Pure Chemical, Osaka, Japan). The double-infected NS/PC-transplanted mice were imaged at week 6. ESAL-NS/PC-transplanted mice were imaged in weeks 6 and 9 or in week 10–11 under inhalation anaesthesia (2% isoflurane and oxygen) or under a mixture of three types of anaesthesia (5 mg/kg butorphanol, 0.75 mg/kg medetomidine hydrochloride, and 4 mg/kg midazolam)^75^ for 9 h, followed by inhalation of 2% isoflurane and oxygen (the luminescent measurements were assessed at weeks 3, 6, and 9 without 9 h of anaesthesia). In each series of experiments, the measurements were performed at the same hour of a day for all mice. For hM3Dq activation, in all animals, CNO solution was injected 7 h before measurement. The signal was measured for 15 min after 50 μl of AkaLumine-HCl (60 mM) and saline solution had been intraperitoneally injected. The region of interest (ROI) was set immediately above the cervical cord, and the peak intensity, observed at approximately 10 min in most cases, was recorded. The measurement parameters were as follows: in vitro; exposure time = 1 s, binning = 8, field of view = 13.4 cm, and f/stop = 1; in vivo; exposure time = 60 s, binning = 8, field of view = 23 cm, and f/stop = 1. All the images were processed with Living Image software (IVIS Imaging Systems, version 4.5.5), and the signal intensity is expressed as the photon count in units of photons/sec*ond*/cm^2^/st*eradian*. Each result is displayed as a pseudocoloured photon count image superimposed on a greyscale anatomic image.

### In vitro luminescence measurement in mouse neurons over time

Hippocampus tissues isolated from E17 mouse embryos were dissected into small pieces and digested with 10 units/ml papain (Nacalai Tesque, Tokyo, Japan) and 0.01% DNase I (Sigma–Aldrich) in PBS at 37 °C for 15 min. Then, hippocampal neurons were cultured (3 × 10^5^ cells) on poly-L-lysine-coated 24-well plates in Neurobasal Plus Medium containing B27 Plus and GlutaMAX. The cells were infected with lentivirus-E-SARE-Venus-AkaLuc-PEST on DIV5, silenced with TTX (1 µM, Tocris, Bristol, UK) on DIV7 and then stimulated with 4AP (250 µM, Tocris) and bicuculline (50 µM, Sigma–Aldrich) in the absence of TTX for 10 min on DIV8 following the methods of previous studies^13,76^. After stimulation, the neurons were silenced again with medium containing 1 μM TTX to suppress prolonged stimulation. At designated time points (0, 2, 4, 6, 8, 10, and 24 h after brief stimulation), bioluminescence images were acquired using the IVIS Spectrum system immediately after treatment with 300 µM AkaLumine-HCl.

### qPCR

Total RNA was extracted by using an RNeasy Micro Kit (Qiagen, Inc., Hilden, Germany), and cDNA was synthesized by reverse transcription with ReverTra Ace qPCR RT Master Mix (Toyobo Co., Ltd., Life Science Department, Osaka, Japan). qPCR was performed using a Step One Plus instrument (Applied Biosystems, CA, USA) following the manufacturer’s instructions. The expression levels of each gene were normalized to those of *ACTB* using the comparative ΔΔCT method. We used the following manufactured primers (Thermo Fisher Scientific) against human DNA sequences: *FOS* (Hs01119266_g1), *ARC* (Hs01045540_g1), and *ACTB* (Hs03023943_g1). Additionally, *EGFP* (Mr00660654_cn) was used to detect Venus expression.

### Immunostaining

In vitro-cultured cells were fixed with 4% paraformaldehyde (PFA) for 15 min. All mice were deeply anaesthetized and transcardially perfused with 4% PFA 10 weeks after injury. Brain and spinal cords were dissected and postfixed in 4% PFA for 2 days. Then fixed spinal cords were soaked in 10% sucrose in 0.1 M PBS overnight at 4 °C, followed by 30% sucrose. The dissected spinal cords were embedded in optimal cutting temperature compound (Sakura Finetek, Tokyo, Japan) and sectioned in the axial plane at a thickness of 12 μm on a cryostat (Leica Biosystems, Wetzlar, Germany). The samples were stained with the following primary antibodies: anti-GFP (goat IgG, 1:500, Rockland, PA, USA), anti-mCherry (rabbit IgG, 1:400, Abcam, Cambridge, UK), anti-panembryonic lethal abnormal vision-like (ELAVL) (mouse IgG1, 1:200, Sigma–Aldrich), anti-GFAP (rabbit IgG, 1:2000, Proteintech, IL, USA), anti-APC (mouse IgG2b, 1:300, Abcam), anti-human GFAP (mouse IgG1, 1:2000, Takara Bio), anti-CNPase (mouse IgG1, 1:2000, Sigma–Aldrich), anti-Ki-67 (rabbit IgG, 1:2000, Leica Biosystems), anti-Nestin (rabbit IgG, 1;200, IBL, Gunma, Japan), anti-HNA (m*ouse* IgG1, 1:100, Millipore, Darmstadt, Germany), anti-Fos (rabbit IgG, 1:400, Abcam), anti-human pan-ELAVL (human IgG, 1:1000, a gift from Dr. Robert Darnell, The Rockefeller University, NY, USA), anti-synaptophysin (mouse IgM, 1:100, Millipore), anti-pan-AMPAR (Guinea pig, 1:500, Frontier Institute, Hokkaido, Japan), and anti-STEM121 (m*ouse* IgG1, 1:200, Takara Bio). The nuclei were stained with Hoechst 33258 (10 μg/ml, Sigma–Aldrich). All images were obtained using a fluorescence microscope (BZ-X710; Keyence, Osaka, Japan/THUNDER Imager Live Cell; Leica, Germany) or confocal laser scanning microscope (LSM 780; Carl Zeiss, Jena, Germany).

### Quantitative analyses of the tissue sections

Quantitative analyses of the tissue sections following SCI and transplantation were performed^35^. Three-dimensional analyses of the Venus+ volume (graft activity)/HNA+ volume (human cells) were performed as follows. Axial sections were prepared from eight animals that underwent ESAL-transduced NS/PC transplantation and anterograde labelling at four sites (4 mice were sacrificed 7 h after CNO administration, and 4 mice were sacrificed without CNO), and the Venus+ area and HNA+ area were determined using ImageJ. The volume was then calculated by the following equation:

1 $${{{{{\rm{V}}}}}}=\frac{h}{3}(A1+\sqrt{A1A2}+A2)$$

where *A*1 and *A*2 are the areas of two consecutive sections and *h* is the distance between them (480 μm).

Quantification of the synapse between CST axons and grafted neurons was performed in combination with a customized macro using ImageJ (ver. 2.1.0/1.53.c). The number of presynaptic markers (synaptophysin) merged with mCherry-labelled CST axon terminals integrated into the STEM121+ area was counted.

### Double immunoelectron microscopy

The detailed procedure used for pre-embedding immunoelectron microscopic analyses has been described previously^77^. Briefly, frozen spinal cord sections on glass slides were thawed, dried and autoclaved in citrate acid buffer (pH 6.0) before blocking treatment (5.0% Block Ace [DS Pharma Biomedical, Osaka, Japan] solution with 0.01% saponin in 0.1 M PB). The samples were stained with the primary antibodies anti-GFP (goat IgG, 1:100, Rockland) and anti-mCherry (rabbit IgG, 1:100, Abcam) and the secondary antibodies anti-rabbit biotin (donkey IgG, 1:800, Jackson ImmunoResearch, PA, USA). After PBS wash Alexa Fluor 488—FluoroNanogold™-conjugated rabbit anti-Goat IgG antibody (1:100, Nanoprobes, NY, USA) was applied before staining with Hoechst 33258. We used the following supplements: ABC complex (VECTASTAIN Elite ABC Kit; Vector, CA, USA), TSA Plus biotin (NEL749A001KT; PerkinElmer, MA, USA), SA-Alexa Fluor 555 (1:1000, Thermo Fisher Scientific), SA-HRP (1:100, Vector), and 3,3′-diaminobenzidine (DAB) tablets (FUJIFILM Wako Pure Chemical). Ultrathin sections (80-nm thickness) were prepared with a diamond knife, collected on copper mesh grids (#100 or #150 Veco, Nisshin EM, Tokyo, Japan), and stained with uranyl acetate and lead citrate in plastic tubes for 10 min each. The sections were examined with a transmission electron microscope (TEM, JEM-1400Plus, JEOL, Tokyo, Japan) at 100 keV.

### Statistics and reproducibility

For comparisons between two groups, a two-tailed Student’s *t* test was used. A repeated-measures ANOVA was used for analyses of the data in Fig. 2h and Supplementary Fig. 4a, c. Two-way repeated-measures ANOVA was used for analyses of the data in Supplementary Fig. 2a–c. For all statistical analyses, differences were considered significant at *p* < 0.05. All data are presented as the mean ± SEM. For all calculations, SPSS Statistics (Japan IBM, Tokyo, Japan. ver. 26) was used. SAS software (SAS Institute, NC, USA. ver. 9.4) was also used for repeated-measures ANOVA. Since this was an exploratory study, we made use of a modest number of samples that may not have been adequate. The appropriate sample size was calculated utilizing the CRAB SWOG Statistical Tools Calculators (https://stattools.crab.org). The primary endpoint of the study was the alteration of graft BLI photon count from pre- to post-CST activation at 10 weeks after TP (Fig. 4l). A post hoc power analysis performed with the dataset in Fig. 4l found that *n* = 11 transplanted mice were sufficient to meet the primary endpoint with a power of 0.8 (One Arm Normal). Cohen’s d was calculated to measure effect size. We define the effect size as large if the *d* value was >0.8^78,79^. The sample sizes of the PBS group and sham group in Supplementary Fig. 2 were determined before the study, with the grip strength test used as the primary endpoint. The a priori power analysis revealed that *n* = 27 total mice were sufficient with a power of 0.8 (Two Arm Normal). The IBB and horizontal ladder analyses were considered secondary.

### Reporting summary

Further information on research design is available in the Nature Research Reporting Summary linked to this article.

## Supplementary information

Supplementary Information

Description of Additional Supplementary Files

Supplementary Data 1

Reporting Summary

## Data Availability

Source data have been included as Supplementary Data 1. The remaining data that support the findings are available from the corresponding authors upon reasonable request.
