# Supplementary material for: A non-invasive system to monitor in vivo neural graft activity after spinal cord injury
Source: Commun Biol. 2022 Aug 10;5:803. doi: 10.1038/s42003-022-03736-8 (PMC9365819; doi:10.1038/s42003-022-03736-8)
Supplement: Supplementary file 1 — Supplementary Information [file 42003_2022_3736_MOESM1_ESM.pdf]

Supplementary Information

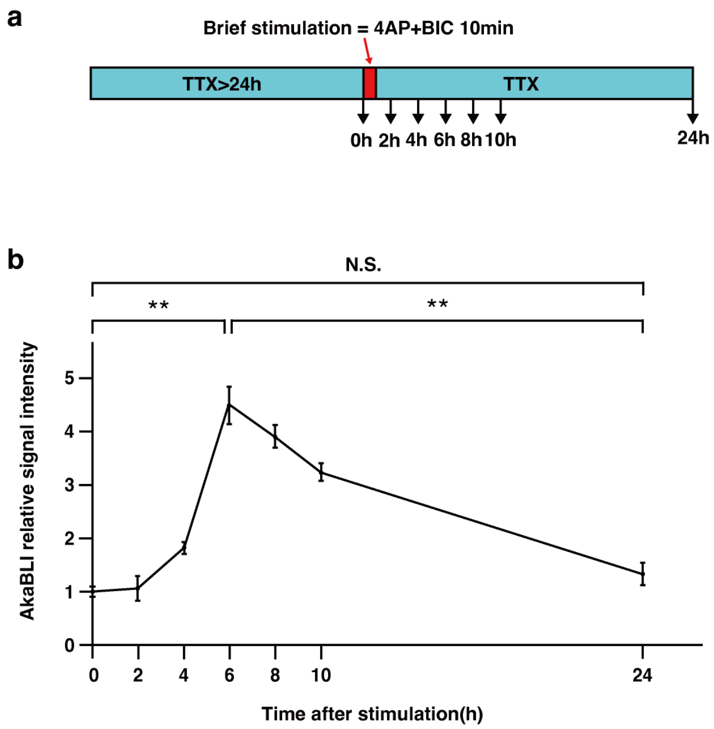

Supplementary Fig. 1 BLI of hippocampal neurons stimulated with 4AP+BIC *in vitro*

(a) Scheme of the experiment performed to detect the time course of ESAL expression using mouse hippocampal neurons. Before and after brief stimulation with 4–aminopyridine (4AP) + bicuculline (BIC), the neurons were silenced with 1 μM TTX to suppress prolonged stimulation. **(b)** Multiple time-point BLI measurements in ESAL-expressing mouse hippocampal neurons. N = 5 independent experiments for 0, 6, and 24 hours, and n = 3 for 2,

4, 8, and 10 hours. The values are the mean ± SEM; *$p < 0.05$, **$p < 0.01$. Two-sided unpaired Student's t tests were performed. Individual t values and degrees of freedom: 0 and 6 hours; $t(8) = 10.42$, $p = 6.2\times10^{-6}$, 6 and 24 hours; $t(8) = 8.357$, $p = 3.2\times10^{-5}$, 0 and 24 hours; $t(8) = 1.568$, $p = 0.16$.

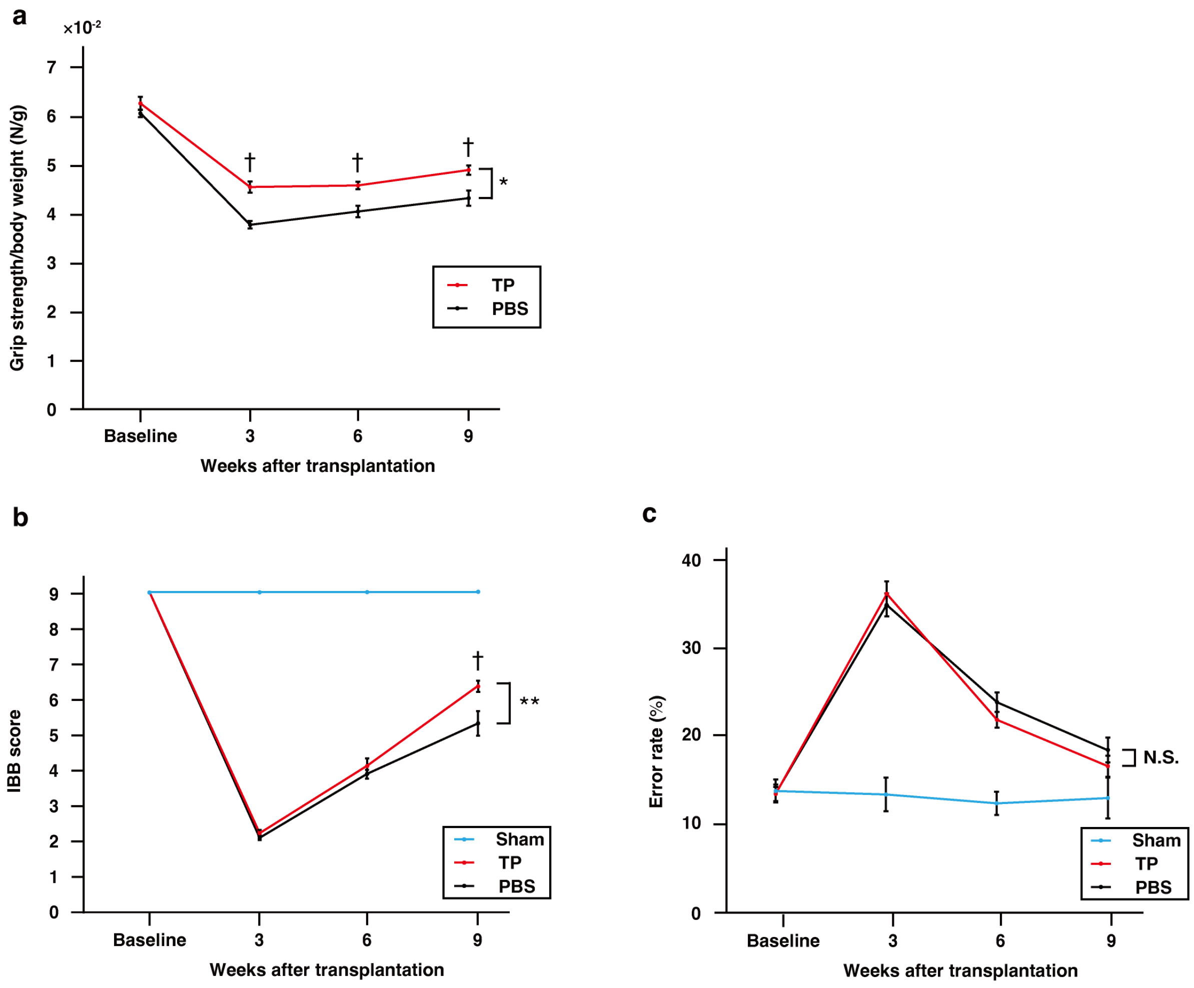

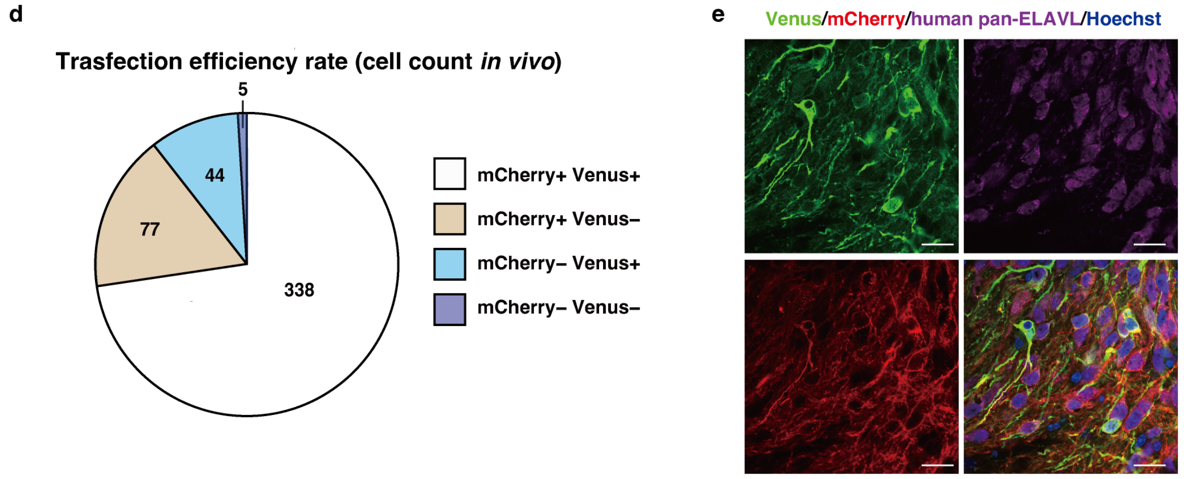

Supplementary Fig. 2 Transplanted NS/PCs promote functional recovery

(a) Grip strength divided by body weight in the TP group and PBS group before and after SCI (TP group, n = 14; PBS group, n = 10). The values are the mean ± SEM: *p < 0.05. Repeated-measures two-way ANOVA was performed; p=0.045. †p < 0.1 according to the Bonferroni correction (Baseline, p = 0.424; week 3, p < 0.002; week 6, p = 0.002; week 9, p = 0.006). **(b)** Food manipulation task. Average IBB scores in the TP group and PBS group before and after SCI (TP group, n=14; PBS group, n=10; sham group, n=4). The values are the mean ± SEM: *p < 0.05. Repeated-measures two-way ANOVA, p=0.008. †p < 0.1 according to the Bonferroni correction (week 3, p = 0.576; week 6, p = 0.830; week 9, p = 0.008). **(c)** Horizontal ladder walking task. Rates of errors made by mice in the TP group and PBS group while walking across the horizontal ladder (TP group, n=14; PBS group, n=10; sham group, n=4).

The values are the mean ± SEM: $*p < 0.05$. Repeated-measures two-way ANOVA, $p=0.099$. **(d)** Quantification of the overlap between the mCherry and Venus proteins. The numbers written on the pie chart represents cell count per 15 images from n = 3 animals. **(e)** Representative images of a positive control mouse (CAG-hM3Dq-mCherry and ESAL-double-transduced NS/PC-transplanted mice); labelled with Venus (green), mCherry (red), and human pan-ELAVL (magenta). Scale bars, 20 μm.

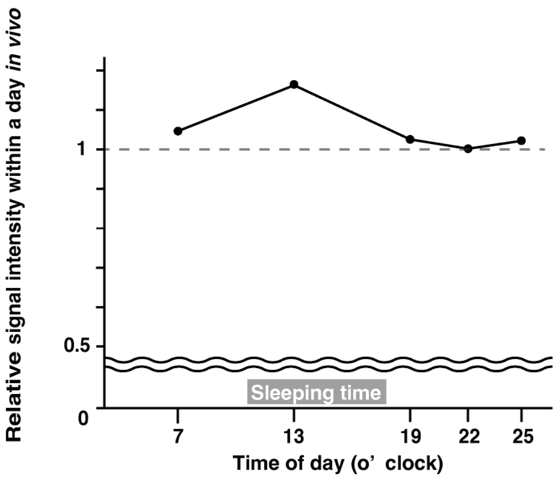

Supplementary Fig. 3 *In vivo* monitoring of ESAL bioluminescence over the day

Representative diurnal transition of luminescence intensity in the cervical spine of an ESAL-NS/PC-transplanted mouse (hM3Dq [-] TP) from Fig. 2b (photons/sec). All measurements

were performed within a week (10–11 weeks after transplantation). The sleeping time in the figure shows a typical example because mice are nocturnal.

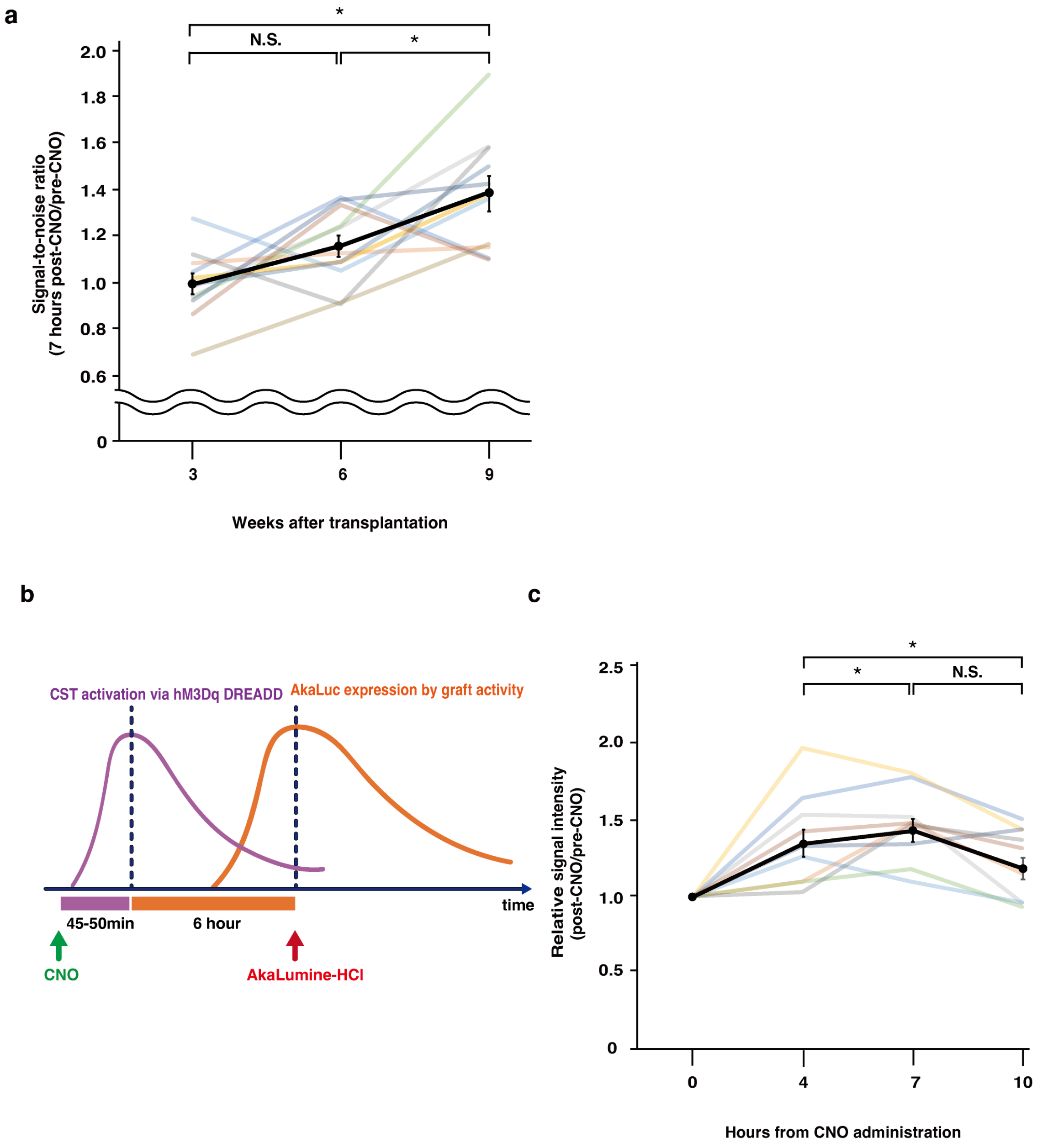

Supplementary Fig. 4 Time course of ESAL activity *in vivo*

(a) The thick line is the average time course of the photon count ratio 3, 6, and 9 weeks after CNO administration *in vivo* for all mice with CST stimulation by DREADD. Post = 7 hours after CNO administration. The thin lines indicate data from individual mice (transplanted mice, n=11). The values are the mean ± SEM: *p < 0.05. Repeated-measures ANOVA was performed; individual p values: 3 and 6 hours; p= 0.053, 3 and 9 hours; <0.0001, 6 and 9 weeks; 0.0072. **(b)** Schematic illustration representing the time course of the *in vivo* experiments after CNO administration. The measurements were consistently performed 7 hours after CNO administration by intraperitoneal injection, when we considered that the AkaLuc photon count had approximately comes to its peak considering the results in Supplementary Fig. 1b. **(c)** The thick line is the average time course of the photon count ratio 4, 7, and 10 hours after CNO administration per 0 hours *in vivo* for all mice with CST stimulation by DREADD in weeks 10–11. The thin lines indicate data from individual mice (transplanted mice, n=10). The values are the mean ± SEM: *p < 0.05. Repeated-measures ANOVA, individual p values: Hour 4 vs. Hour 0 and Hour 7 vs. Hour 0; 0.2132, Hour 4 vs. Hour 0 and Hour 10 vs. Hour 0; 0.0310, Hour 7 vs. Hour 0 and Hour 10 vs. Hour 0; 0.0018. Two-sided paired Student's t test, individual t values and degrees of freedom: Hour 0 and

Hour 4; t (18) =2.98, p=0.0079, Hour 0 and Hour 7; t (18) = 3.73, p =0.0015, Hour 0 and Hour 10; t (18) = 0.783, p = 0.4435.

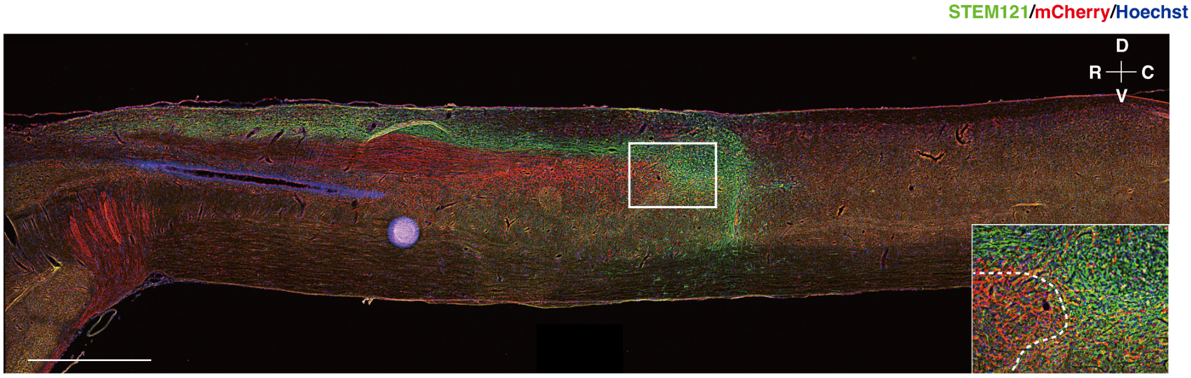

Supplementary Fig. 5 Immunohistochemical image showing host-to-graft connectivity

Representative overall zoomed out image near the sagittal section shown in Fig. 4c. Transplanted cells were stained with STEM121 (a human-specific cytoplasmic marker), and the CST was labelled with mCherry. Dashed line in the enlarged image, rostral boundary of the graft, R: rostral, C: caudal, D: dorsal, and V: ventral side. Scale bars, 1 mm.

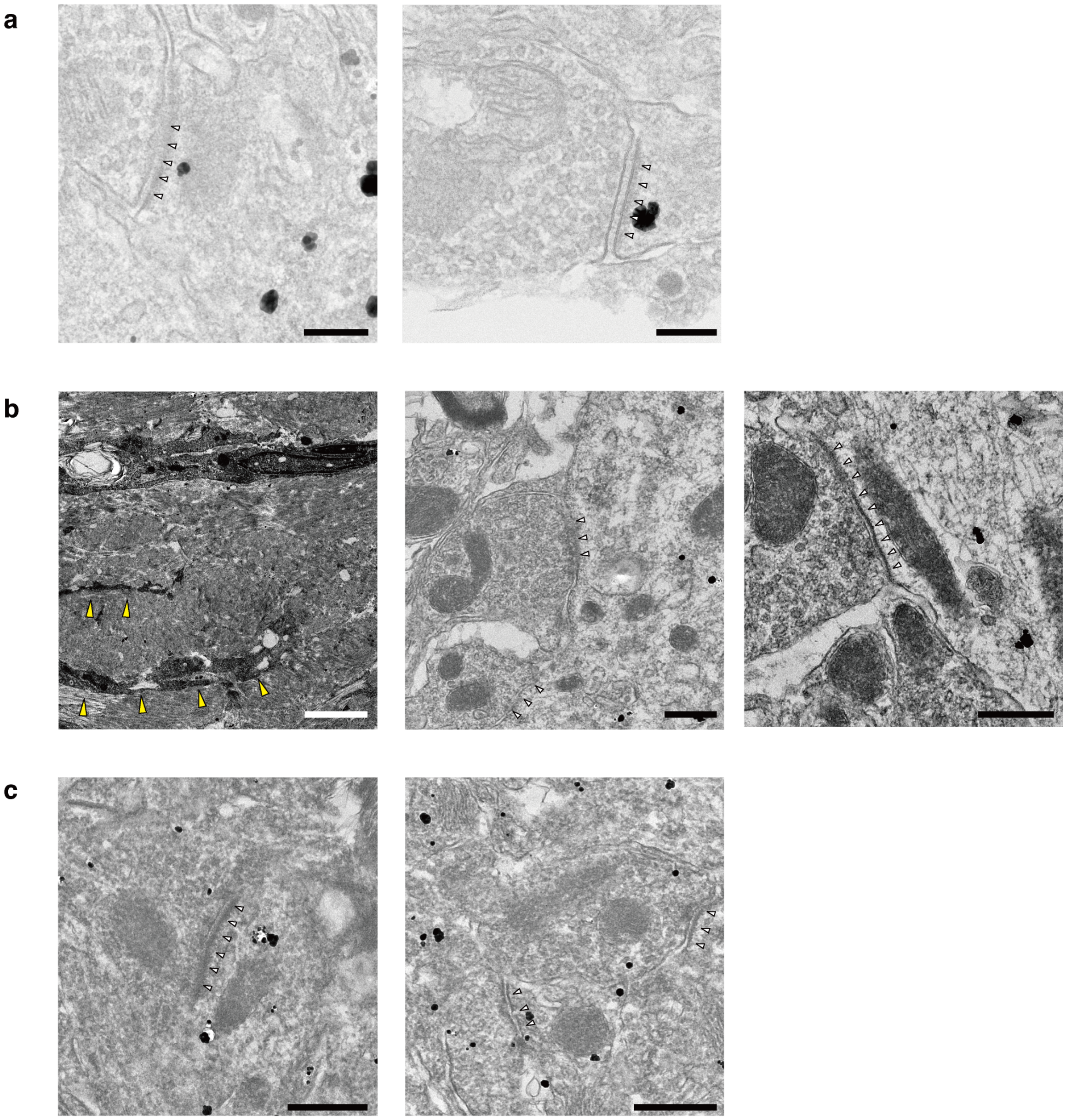

Supplementary Fig. 6 Comparison with immunoelectron microscopic images of staining of another tissue as a negative control

(a) Immunoelectron microscopic image of a synaptic connection without 3,3'-diaminobenzidine tetrahydrochloride (DAB) staining as a negative control. Only Venus+ graft

neurons are stained by Fluorogold. Arrowheads, postsynaptic density. Scale bar, 200 nm. **(b)** Double immunoelectron microscopic images of the synaptic connection between an mCherry+ CST neuron with 3,3'-diaminobenzidine tetrahydrochloride (DAB) staining and a Venus+ graft neuron with Fluorogold staining under the same conditions as in Fig. 4g. Low magnification. Arrowheads, CST axons with DAB+. Scale bars, 2 μm (left). High-magnification view of synaptic connections between a CST neuron and a graft neuron detected around the axonal ends of CST neurons (middle, right). Arrowheads, postsynaptic density. Scale bars, 200 nm, 500 nm. **(c)** High-magnification view of a synaptic connection between Venus+ graft neurons with Fluorogold staining in another area of Supplementary Fig. 1b. Arrowheads, postsynaptic density. Scale bars, 500 nm.
